# Lipases as Tools in the Synthesis of Prodrugs from Racemic 9-(2,3-Dihydroxypropyl)adenine

**DOI:** 10.3390/molecules171213813

**Published:** 2012-11-22

**Authors:** Jana Brabcová, Jiří Blažek, Lucie Janská, Marcela Krečmerová, Marie Zarevúcka

**Affiliations:** 1 Institute of Chemical Technology Prague, Faculty of Food and Biochemical Technology, Technická 5, 160 28 Prague 6, Czech Republic; Email: brabcova@uochb.cas.cz (J.B.); blazek@uochb.cas.cz (J.B.); lucie.janska@seznam.cz (L.J.); 2 Institute of Organic Chemistry and Biochemistry AS CR, Flemingovo n. 2, 166 10 Prague 6, Czech Republic; Email: krecmerova@uochb.cas.cz

**Keywords:** lipase, transesterification, prodrug, immobilization of enzymes

## Abstract

Lipases from *Geotrichum candidum* 4013 (extracellular lipase and cell-bound lipase) were immobilized by adsorption on chitosan beads. The enzyme preparations were tested in the synthesis of ester prodrugs from racemic 9-(2,3-dihydroxypropyl)adenine in dimethylformamide with different vinyl esters (acetate, butyrate, decanoate, laurate, palmitate). The transesterification activities of these immobilized enzymes were compared with commercially available lipases (lipase from hog pancreas, *Aspergillus niger*, *Candida antarctica*, *Pseudomonas fluorescens*). Lipase from *Candida antarctica* was found to be the most efficient enzyme regarding chemical yield of the desired products, while transesterification by lipase from *Aspergillus niger* resulted in lower yields.

## 1. Introduction

Lipases, (triacylglycerol acylhydrolases; EC 3.1.1.3.) are one of the most important classes of hydrolytic enzymes. Their enantio-, chemo- and stereo-selective nature makes them useful tools in the field of organic synthesis. 

Lipases are known to be highly thermostable and active, even when an organic solvent is used as the reaction medium [1]. In comparison with traditional chemical methods, the main advantage of using enzymatic synthesis in the preparation of prodrugs arises from the possibility of performing reactions under milder conditions, thus making it possible to utilize combinatorial enzymology as an alternative to standard approaches in combinatorial chemistry. 

The lipase-catalyzed formation of esters from alcohols and ester acyl donors (transesterification) is a reversible reaction. The use of activated esters as acyl donors shifts the equilibrium constant in favor of the product. Several activated esters have been developed, but the most useful acyl donors are enol esters such as vinyl esters [2,3,4]. Lipases are often used in the synthesis of prodrugs, because of their transesterification activity. Esters are the most common type of prodrugs that are converted back to the active parent compounds via the ubiquitous esterases present in blood, tissues and organs [5,6].

The aim of this work was to perform the screening for a suitable biocatalyst for the synthesis of ester prodrugs from racemic 9-(2,3-dihydroxypropyl)adenine (DHPA) in dimethyl formamide. For this reason, the catalytic activity of five commercially available lipases (lipase from hog pancreas, *Aspergillus niger*, *Candida antarctica*, *Pseudomonas fluorescens*, *Mucor miehei*) and two immobilized lipases from *G. candidum* 4013 were tested. Enantioselectivity and regioselectivity of both lipases produced from *Geotrichum candidum* 4013 have been already studied [7,8].

The selected starting compound, 9-(2,3-dihydroxypropyl)adenine, belongs to the group of acyclic nucleoside analogues, compounds whose structural attribute is a nucleobase attached to a polyhydroxylic carbon chain. Several representatives of this group (acyclovir, ganciclovir, penciclovir) are approved antiviral agents with activity targeted against herpeviruses. The most potent agents were found to be *N*^9^-alkyl derivatives of adenine with hydroxyl group(s) on the alkyl chain: (*R*,*S*)-3-(adenin-9-yl)-2-hydroxypropanoic acid (AHPA and its alkyl esters), D-eritadenine and the broad-spectrum antiviral agent (*S*)-(2,3-dihydroxypropyl)adenine [9,10,11,12]. 

The selected starting compound, 9-(2,3-dihydroxypropyl)adenine, belongs to the group of acyclic nucleoside analogues, compounds whose structural attribute is a nucleobase attached to a polyhydroxylic carbon chain. Several representatives of this group (acyclovir, ganciclovir, penciclovir) are approved antiviral agents with activity targeted against herpeviruses. The most potent agents were found to be *N*^9^-alkyl derivatives of adenine with hydroxyl group(s) on the alkyl chain: (*R*,*S*)-3-(adenin-9-yl)-2-hydroxypropanoic acid (AHPA and its alkyl esters), D-eritadenine and the broad-spectrum antiviral agent (*S*)-(2,3-dihydroxypropyl)adenine [9,10,11,12]. 

The antiviral potency of these adenosine analogues results from their inhibition of SAM-dependent methylation reactions via inhibition of *S*-adenosylhomocysteine (SAH) hydrolase. The most effective inhibitor of SAH hydrolase, DHPA, is an approved drug for the topical treatment of herpes labialis (HSV-1) in the former Czechoslovakia, marketed under the name Duvira^®^ gel.

Very low oral bioavailability of all acyclic nucleoside analogues is a limitation for their use as topical drugs only. Thus far, the only oral prodrugs used clinically are valganciclovir (valine prodrug of ganciclovir) and famciclovir (diacetyl derivative of penciclovir). Development of new methodologies for the transformation of acyclic nucleoside analogues into their ester prodrugs is thus highly desirable. 

## 2. Results and Discussion

### 2.1. Selection of Lipases

**Scheme 1:**
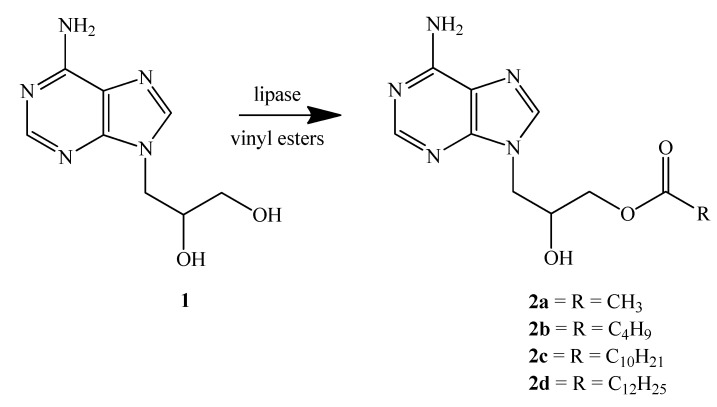
Reaction pathway.

The selection of effective enzymes for the synthesis of ester prodrugs from racemic 9-(2,3-dihydroxypropyl)adenine in dimethyl formamide (Scheme 1) was performed following a well established screening procedure. The active and selective enzymes, lipases from *Pseudomonas fluorescens* (free form), *Candida antarctica* (free form), *Aspergillus niger* (free form), hog pancreas (free form) and *Geotrichum candidum* 4013 (cell-bound lipase as the acetone powder, immobilized cell-bound lipase and immobilized extracellular lipase) were used for preparative scale purposes. 

It was previously described that lipase from *Aspergillus oryzae* had a higher specificity toward triacylglycerols of medium-chain saturated fatty acids over short-chain or long-chain fatty acids. The enzyme demonstrated 1,3-positional specificity [13]. Berger and Schneider [14,15] developed a facile system for the quantitative determination of lipase regioselectivities in organic solvents towards the 1,3 position of glycerides. Their study showed that the lipases from *Mucor meihei* and *Pseudomonas fluorescens* did not display high 1,3-positional specificities. It was reported that the broad isoelectric region of *Candida antarctica* lipase B is unique as compared to almost all other α/β-hydrolases which have a well-defined isoelectric point [16]. However, for some lipases the chain length of the fatty acid moiety of the ester substrate can have an effect on reaction rate. It was reported that the lipases from *C. rugosa *and porcine pancreas exhibited a preference for shorter-chain substrates, whereas the lipase from *R. miehei *showed little significant difference in transesterification reaction rate with a range of C_8_–C_18_ fatty acid moieties [17].

### 2.2. Lipases from G. Candidum and Their Immobilization

The lipases from *Geotrichum candidum* were used as a biocatalyst in the transesterification of DHPA as well. It was already found that the fungus *Geotrichum candidum *4013 produces two types of lipases (extracellular and cell-bound [7]). The extracellular lipase displays low selectivity against polyunsaturated fatty acids, while the cell-bound lipase possesses selectivity to saturated fatty acids [18]. Enantiomeric ratio (*E*) achieved with both induced enzymes in the tested processes was high (from 43 to 242, [19]). Until now the catalytic activities of the enzymes were tested using acetone powder (cell-bound lipase [20]) or lyophilized powder (extracellular lipase, [20]) and they exhibited excellent enantioselectivity and chemical yields. In the present study we decided to use the enzymes in their immobilized form, because an adequate immobilization procedure can greatly improve and modulate the catalytic properties of lipases like thermostability and selectivity [21]. Another benefit of using immobilized enzyme is reusability of the biocatalysts. Therefore lipases from *G. candidum* 4013 (extracellular and cell-bound) were immobilized to chitosan beads. Chitosan exhibits several advantages as an immobilization carrier, including low biodegradability and easy handling.

Since the amount of enzyme bound to the support, as well as their activity, would be largely influenced by the time and temperature of immobilization, these parameters were optimized initially (data not shown). An immobilization time of 3 h was found to be optimal at 25 °C. Under these conditions, a protein loading from 33% to 48% was obtained. The hydrolytic activity of the immobilized extracellular lipase was 0.052 U/g chitosan beads (Table 1) and the activity of immobilized cell-bound lipase was 2.5 higher in comparison to the activity of immobilized extracellular lipase. 

**Table 1:** Yields of protein loading and activity of lipase immobilized to chitosan beads.

Enzyme	Protein content of free lipase solution before immobilization (mg/mL)	Protein content of free lipase solution after immobilization (mg/mL)	Protein loading yield (%)	Specificactivity of lipase solution before immobilization (U/mg protein)	Specific activity of immobilized lipase (U/g chitosan beads)
Extracellular lipase	2.82	1.86	33	0.145	0.052
Released lipase	9.16	4.70	48.6	0.291	0.125

### 2.3. Enzymatic Transesterification

It is known that the nature of the solvent and the type of nucleophile can significantly influence the enzymatic esterification. Herein dimethylformamide has been chosen as the best solvent because of the low solubility of DHPA in the other commercially available solvents. The effect of chain length of the fatty acid moiety of vinyl esters on lipase activity was tested. Generally, the chemical yield of the reaction decreased when acyl donors with a higher chain length were used (Table 2). The only exception was observed when lipase from *P. fluorescens* was used as biocatalyst in transesterification between vinyl butyrate and DHPA. Under these conditions the highest chemical yield among all obtained results was 72%. In the case of vinyl palmitate all of the tested enzymes were catalytically inactive.

Sample composition was determined by HPLC performed on a Waters 600E system. By this analytical method any remaining DHPA leaves the column first, with a retention time of 5.1 min, followed by monoacylated compounds with retention times ranging from 7 to 16 min (**2a** = 7.2 min, **2b** = 10.2, **2c** = s 13.7 min and **2d** = 15.7 min) at 9.3 ± 0.2 MPa. Diacylated compounds are supposed to be formed, but we determined only monoacylated mixtures of two such products. Diols, which contain secondary and primary hydroxy groups, can be selectively monoacylated at the primary hydroxy group by many lipases in organic solvents. Since the reaction does not take place at the chiral secondary center itself, observed enantioselectivities are usually low or enantioselectivity was not observed at all [22]. Usually this kind of reaction proceeds with very low (or none) enantioselectivity, although some specific classes of primary alcohol can be acylated with high enantioselectivity by mean of lipase catalysis [23,24,25]. The phenomenon that diols are inferior substrates than their corresponding monoacylated derivatives in lipase catalyzed transesterification is generally known. It was also observed for racemic diols with C2 symmetry [26,27] where the first acylation step is less enantioselective than the second one. An analogous behavior was found for many prochiral diols [28,29,30,31] in which the monoacylation step shows a low or only moderate enantioselectivity, but in many cases the low enantioselectivity can be overcome by application of a sequential lipase-catalyzed acylation procedure [22].

**Table 2:** Chemical yields of transesterification catalysed by lipases (the reaction was repeated twice using two independent synthetic procedures. The average values of the rates are reported in the Table).

Source of lipase	Vinyl ester *	Chemical yield (%)
*Geotrichum candidum–*acetone powder	VA	49
	VB	37
	VD	30
	VL	28
*Geotrichum candidum–*chitosan beads (extracellular lipase)	VA	25
	VB	20
	VD	17
	VL	8
*Geotrichum candidum–*chitosan beads (cell-bound lipase)	VA	20
	VB	13
	VD	13
	VL	10
*Candida antarctica*	VA	66
	VB	46
	VD	40
	VL	25
*Aspergillus niger*	VA	42
	VB	40
	VD	38
	VL	30
*Pseudomonas fluorescens*	VA	51
	VB	72
	VD	49
	VL	42
Hog pancreas	VA	58
	VB	49
	VD	36
	VL	28

* VA-vinyl acetate, VB-vinyl butyrate, VD-vinyl decanoate, VL-vinyl laurate.

Satisfactory chemical yields were obtained with lipase from *Candida antarctica* and lipase from *Pseudomonas fluorescens* when they were used as biocatalysts (Table 2). In the case where acetone powder of *Geotrichum candidum* cells was used as biocatalyst the chemical yield of the transesterification was comparable with results obtained using commercially available enzymes. On the other hand, both lipases from *Geotrichum candidum *4013 immobilized on chitosan beads provided less catalytic activities than the free forms. Generally it is known that the immobilization of enzymes has an effect on the catalytic properties of enzyme. Various methods of immobilization of lipases on many different types of supports are available. Our experimental investigations have produced unexpected results, such as a significant reduction in lipase activity compared with the acetone powder form of the enzyme. A more detailed search for a convenient support for the immobilization of lipases will be the most critical point for resolving this problem. Other key components the type of support used for immobilization, conjugation chemistry, conjugation conditions, and selection of reaction a medium all have complex effects on the enzyme activity for a particular substrate and will be optimized accordingly.

### 2.4. Reusability of Immobilized Lipases from G. candidum

One of the important characteristics of an immobilized enzyme is its stability and reusability over an extended period of time. The reusability of both the immobilized lipase from *G. candidum* was evaluated in the transesterification of DHPA (see Experimental section) at 25 °C and 70% of the initial activity was retained after 2 runs. Between cycles, the catalysts were washed with dry hexane and DMF and thereafter used without drying. Our results coincide with results previously published [32,33]. The repeated use of the immobilized *P. chrysogenum* lipase in the hydrolysis of olive oil was studied. After six reuses, immobilized lipase retained 72.09% of its activity [34]. Hung and coworkers found that with repeated use immobilized *C. rugosa* lipase on chitosan retained 74% after 10 reuses [32]. Similarly Yi and coworkers showed that the activity of immobilized lipase of *C. rugosa *on alanine chitosan beads retained 77% of the initial activity after 10 reuses [35]. Antiviral activities of all the above described DHPA prodrugs is currently being investigated by the team of Professor Jan Balzarini at the Rega Institute for Medical Research KU Leuven (Belgium).

## 3. Experimental

### 3.1. Microorganism and Chemicals

The strain of *Geotrichum candidum* 4013 was obtained from the Culture Collection of the Department of Biochemistry and Microbiology (DBM), Institute of Chemical Technology, Prague. Lipases [hog pancreas (2.4 U/mg), *Aspergillus niger* (4 U/g), *Candida antarctica* (3.0 U/mg) *Pseudomonas fluorescens* (42.5 U/mg)] and chemicals were purchased from Sigma Aldrich (St. Louis, MO, USA). Racemic 9-(2,3-dihydroxypropyl)adenine (DHPA) was prepared in the laboratory of Dr. Krečmerová.

### 3.2. Preparation of Lipases from Geotrichum candidum 4013 [19]

For solid culture, *Geotrichum candidum* 4013 was inoculated from culture slant. Growth medium with the following composition per liter was used: glucose (30 g), corn steep (10 g), MgSO_4_·7H_2_O (0.5 g), KH_2_PO_4_(1 g), K_2_HPO_4_(1 g), NaNO_3_(2 g), KCl (0.5 g), FeSO_4_·7H_2_O (0.02 g) and agar (15 g). The tubes were incubated at 25°C for 3 days and conserved at 4 °C. Liquid culture was prepared with medium consisting of glucose (30 g), corn steep (10 g), MgSO_4_·7H_2_O (0.5 g), KH_2_PO_4_(1 g), K_2_HPO_4_(1 g), NaNO_3_(2 g), KCl (0.5 g), FeSO_4_·7H_2_O (0.02 g) in a simple conical flask (250 mL) containing 100 mL of the medium and closed with sterile stopcocks. The medium was sterilized at 121 °C for 20 min. The medium was inoculated with the cells originally growing on culture slants and incubated with shaking at 30 °C for 24 h. For the activation of the lipase production (extracellular and cell-bound) the medium containing per litre: peptone (50 g), glucose (10 g), MgSO_4_·7H_2_O (1 g), NaNO_3_(1 g), olive oil (10 g) was used. Medium (90 mL) was inoculated with 10 mL of prepared inoculum. After cultivation the broth was filtered. Cell pellet was used as source of cell-bound lipase and supernatant as crude extracellular lipase.

Cells were harvested by filtration of 100 mL cell suspension (inoculated medium) through a 0.2 µm filter, the remaining eluate was washed in distilled water (100 mL). The cells of *G. candidum* 4013 (3 g wet wt) were mixed with cold acetone (−20 °C, 100 mL), and the cells were collected by filtration. The procedure was repeated five times, and then the cells were dried under atmospheric pressure and ambient temperature. The dried cells (0.5 g) were obtained and used without further purification as crude enzyme (acetone powder) and refrigerated under nitrogen for storage.

### 3.3. Extraction of Enzyme from the Cell Wall—Preparation of Released Lipase [8,21]

The cell pellet was separated from the broth (100 mL) by filtration and washed by ice-chilled acetone (200 mL). The suspension was filtrated and the pellet was dissolved in 200 ml of acetone (−20 °C). The suspension was vigorously stirred 2 h. After filtration of the mixture, the pellet was dissolved in 200 ml of diethyl ether (−20 °C), filtered and dried at room temperature on the filter. Enzyme was extracted from the cells by 0.05 M Tris HCl buffer pH 8.0 (5 mL) at room temperature with constant stirring for 90 min. The cell free extract was obtained by filtration. The crude extract containing free lipase in the aqueous phase was stored at 4 °C.

### 3.4. Immobilization of Lipases On Chitosan Beads [19,35]

Preparation of chitosan beads: 3% (w/v) chitosan powder was dissolved in 1% acetic acid. Spherical beads of diameter in the range 1–2 mm were produced by adding the chitosan solution dropwise into a coagulant bath consisting of 1M NaOH containing 26% (v/v) ethanol under stirring. Allowing the mixture to remain overnight, the spherical beads were removed by filtration and washed with deionized water until neutrality. The beads then were stored in deionized water at 4 °C until use. Chitosan beads (18 g) had been previously soaked in hexane under agitation for 1 h. Then, excess hexane has removed followed by the addition of 60 mL of crude extracelular lipase (supernatant, concentration 1.86 mg protein/mL) or 60 mL of the crude extract containing free lipase in the aqueous phase (4.70 mg protein/mL). The mixture was agitated for 3 h at room temperature followed by an additional period of 18 h under static conditions at 4 °C. The immobilized lipase on chitosan beats was filtered off, rinsed with hexane and was stored in water at 4 °C until use.

### 3.5. Enzyme Activity Assay

The lipase activity of all used enzymes was determined before our experiments by a spectophotometric method (Table 3). The activity assays of commercial enzymes declared by the supplier were different concerning substrate, pH and temperature.

**Table 3:** Enzymes and their activities.

Lipase from	Activity declared by Sigma ^a^	Activity determination spectrophotometrically ^b^
*Aspergillus niger*	4 U/g	2.1 U/g
*Candida antarctica*	3.0 U/mg	2.62 U/g
*Pseudomonas fluorescens*	42.5 U/mg	1.8 U/g
Hog pancreas	2.4 U/mg	0.569 U/g
*Geotrichum candidum*-acetone powder	-	0.083 U/g

^a^ Unit def.: 1 U corresponds to 1 μmol product/min; ^b^ Unit def.: 1U corresponds to 1 μmol *p*-nitrophenol/min.

The release of yellow *p*-nitrophenol due to hydrolysis of *p*-nitrophenyl laurate by lipase was measured. A reaction mixture containing 3 mM *p*-nitrophenyl laurate (100 µL, dissolved in 2-propanol), 50 mM Tris-HCl (1 mL, pH 7.5), and 50 µL of crude extracellular or free cell-bound lipase or 50 mg of cell-bound lipase as acetone powder or fresh cells (prepared as mentioned above) was incubated at 25 °C. Since autohydrolysis of substrates produced low but significant background values at 410 nm, the absorbance in each assay was measured against a substrate-buffer mixture. The *p*-nitrophenol released was monitored spectrophotometrically at 410 nm. One lipase unit (U) was defined as the amount of enzyme that released 1 µmol *p*-nitrophenol per minute.

### 3.6. Transesterification

An enzyme (0.025 U, commercially available lipases or immobilized lipases prepared according to the paragraph above) was added to a solution of the substrate DHPA (22 mg; 0.1 mmol) in DMF (1.5 mL). The reaction was started by addition of vinyl esters (0.5 mL or 0.2 mmol of vinyl palmitate in 0.5 mL of DMF). The reaction was performed in flasks under stirring at 25 °C for more than 6 days. The progress of the reaction was monitored by TLC analysis. Final work-up consisted of filtration off of the enzyme and evaporation of the solvent, and chromatographic separation of the residue. 

### 3.7. Analytical Methods

The ^1^H-NMR and ^13^C-NMR spectra were recorded in DMSO–*d_6_* on Bruker Avance II 600 and/or Bruker Avance II 500 spectrometers operating at 600.0 or 500.0 MHz in ^1^H and 150.9 or 125.7 MHz in ^13^C. TLC was carried out on precoated silica gel TLC plates. A column (250 mm × 4 mm) filled with a Separon SGX C_18_ solid phase (5 μm; Watrex) was employed for HPLC analysis of sample composition using gradient of Solvent A (water) and Solvent B (acetonitrile): From 100 to 0% Solvent A in the 30 min as mobile phase at 1 mL min^−1^. Detection of the compounds during the HPLC analysis was at 254 nm. Products of the reactions were characterized by ^1^H-NMR and ^13^C-NMR analysis. Optical rotation was measured on Perkin-Elmer 241 polarimeter and mass spectrum was obtained from LTQ Orbitrap XL (Thermo Fisher Scientofic).

#### Preparative TLC

The products of the enzymic reactions were separated into remaining DHPA and esters of DHPA by TLC, and developed in a solvent mixture containing chloroform and methanol (4:1) mixture. The separation was made using DC-Alufolien Kieselgel 60 F254 pre-coated TLC sheeds (Merk) with silica gel layer (0.2 mm). The bands were identified using UV (254 nm). Fractions were extracted from the plates with freshly distilled and dry mixture of chloroform and methanol (4:1), evaporated and weighed to calculate yields. 

*Compound ***2a**: ^1^H-NMR (DMSO–*d_6_*): 8.13 (1H, s, H-2), 8.05 (1H, s, H-8), 7.19 (2H, bs, -NH_2_), 5.49 (1H, d, *J*_OH-2__′_ = 5.0, -OH), 4.23 (1H, m, H-1′b), 4.08–4.14 (2H, m, H-1′a, H-2′), 3.96 (1H, dd, *J*_gem_ = 11.4, *J*_3__′__b-2′_ = 4.4, H-3′b), 3.91 (1H,dd, *J*_gem_ = 11.4, *J*_3__′__a-2′_ = 5.5, H-3′a), 2.00 (3H, s, H-5′). ^13^C-NMR (DMSO–*d_6_*): 170.5 (C-4′), 156.1 (C-6), 152.6 (C-2), 149.9 (C-4), 141.7 (C-8), 118.8 (C-5), 66.7 (C-2′), 65.8 (C-3′), 46.3 (C-1′), 20.9 (C-5′). UPLC/MS (ES^+^), *m/z*: found 252.4 [MH^+^], [α]^24^_*D*_ = 0.

*Compound ***2b**: ^1^H-NMR (DMSO–*d_6_*): 8.12 (1H, s, H-2), 8.05 (1H, s, H-8), 7.19 (2H, bs, -NH_2_), 5.48 (1H, d, *J*_OH-2__′_ = 5.0, -OH), 4.23 (1H, m, H-1′a), 4.08–4.14 (2H, m, H-1′b, H-2′), 3.92–3.99 (2H, m, H-3′), 2.26 (2H, t, *J*_5__′__-6′_ = 7.3, H-5′), 1.53 (2H, sextet, *J*_6__′__-5′_ = *J*_6__′__-7′_ = 7.4, H-6′), 0.88 (3H, t, *J*_7__′__-6′_ = 7.4, H-7′). ^13^C-NMR (DMSO–*d_6_*): 172.8 (C-4′), 156.1 (C-6), 152.5 (C-2), 149.9 (C-4), 141.7 (C-8), 118.7 (C-5), 66.7 (C-2′), 65.7 (C-3′), 46.3 (C-1′), 35.4 (C-5′), 18.0 (C-6′), 13.6 (C-7′). UPLC/MS (ES^+^), *m/z*: found 280.5 [MH^+^], [α]^24^_*D*_ = 0.

*Compound ***2c**: ^1^H-NMR (DMSO–*d_6_*): 8.11 (1H, s, H-2), 8.04 (1H, s, H-8), 7.19 (2H, bs, -NH_2_), 5.48 (1H, d, *J*_OH-2′_ =5.0, -OH), 4.23 (1H, m, H-1′b), 4.06-4.14 (2H, m, H-1′a, H-2′), 3.96 (1H,bdd, *J*_gem_ = 11.4, *J*_3__′__b-2′_ = 4.8, H-3′b), 3.93 (1H,bdd, *J*_gem_ = 11.4, *J*_3__′__a-2′_ = 5.3, H-3′a), 2.27 (2H, t, *J*_5′-6′_ = 7.4, H-5′), 1.50 (2H, pent, *J*_6__′__-5′_ = 7.3, H-6′), 1.17–1.31 (12H, m, H-7′-12′), 0.84 (3H, t, *J*_19′-18′_ = 7.0, H-13′). ^13^C-NMR (DMSO–*d_6_*): 173.0 (C-4′), 156.1 (C-6), 152.5 (C-2), 149.9 (C-4), 141.7 (C-8), 118.8 (C-5), 66.7 (C-2′), 65.7 (C-3′), 46.3 (C-1′), 33.6 (C-5′), 31.5 (C-11′), 28.7–29.1 (C-7′-10′), 24.6 (C-6′), 22.3 (C-12′), 14.2 (C-13′). UPLC/MS(ES^+^), m/z: found 364.6 [MH^+^], [α]^24^_*D*_ = 0.

*Compound ***2d**: ^1^H-NMR (DMSO–*d_6_*): 8.11 (1H, s, H-2), 8.04 (1H, s, H-8), 7.19 (2H, bs, -NH_2_), 5.48 (1H, *J*_OH-2__′_ = 5.0, -OH), 4.23 (1H, m, H-1′b), 4.06–4.14 (2H, m, H-1′a, H-2′), 3.96 (1H, dd, *J*_gem_ = 11.4, *J*_3__′__b-2′_ = 4.6, H-3′b), 3.93 (1H,dd, *J*_gem_ = 11.4, *J*_3__′__a-2′_ = 5.4, H-3′a), 2.27 (2H, t, *J*_5__′__-6′_ = 7.4, H-5′), 1.50 (2H, pent, *J*_6__′__-5′_ = 7.3, H-6′), 1.20–1.28 (16H, m, H-7′-14′), 0.84 (3H, t, *J*_19__′__-18′_ = 7.0, H-15′). ^13^C-NMR (DMSO–*d_6_*): 172.9 (C-4′), 156.1 (C-6), 152.5 (C-2), 149.9 (C-4), 141.7 (C-8), 118.8 (C-5), 66.7 (C-2′), 65.7 (C-3′), 46.3 (C-1′), 33.6 (C-5′), 31.5 (C-13′), 28.7–29.2 (C-7′-12′), 24.6 (C-6′), 22.3 (C-14′), 14.2 (C-15′). UPLC/MS (ES^+^), *m/z*: found 392.6 [MH^+^], [α]^24^_*D*_ = 0.

## 4. Conclusions

In the present preliminary study it was shown that five commercially available lipases were capable of catalysing the transesterification of racemic 9-(2,3-dihydroxypropyl)adenine as shown in Scheme 1. As the reaction site is relatively far removed from the chiral centre, no chiral discriminations were observed using these lipases, even when highly enantioselective lipases from *Geotrichum candidum* 4013 were used as biocatalysts. The lipases were immobilized on chitosan beads. The crucial point of using immobilized lipases for esterification of DHPA is recoverability and reusability with a focus on immobilization of the enzymes in terms of support selection. The easy removal of the enzyme particles from the reaction media offers an important advantage from the economic point of view. These data obtained from analytical scale enzymatic reactions (screening) allowed for further exploration of the optimal conditions for each individual biotransformation concerning chemical yields of the reactions with interest in minimization of the reaction time.
